# Structure-dependent activity of plant natural products against methicillin-resistant *Staphylococcus aureus*

**DOI:** 10.3389/fmicb.2023.1234115

**Published:** 2023-08-15

**Authors:** Calisto Moreno Cardenas, Serhat S. Çiçek

**Affiliations:** Department of Pharmaceutical Biology, Institute of Pharmacy, Kiel University, Kiel, Germany

**Keywords:** MRSA, natural product, antimicrobial resistance, antibiotic, phloroglucinol, sesquiterpenoid, diterpenoid, macrolide

## Abstract

Methicillin-resistant *Staphylococcus aureus* (MRSA) is one of the major causes for nosocomial infections and has been classified as “high priority pathogen” by the World Health Organization. Its ability to develop resistances has been a challenge for the last decades and is still a threat to health care systems, as strains with resistances to the so-called drugs of last resort have been discovered. Therefore, new antibiotics are urgently needed. Natural products are an important source for the development of new drugs, thereby mostly serving as lead compounds for further modification. In this review, the data on plant natural products with reported anti-MRSA activity until the end of 2022 is discussed, highlighting the most effective drugs with respect to their inhibitory concentrations as well as with regard to eventual synergistic effects with existing antibiotics. In the latter sense, the class of alkaloids must be mentioned, exhibiting additive or synergistic effects by inhibiting bacterial efflux pumps. With regard to the antibiotic activity, phloroglucinol derivatives certainly belong to the most promising compounds, revealing several candidates with remarkable effects, e.g., lupulone, ivesinol, rhodomyrtone, aspidinol, or hyperforin. Also, the class of terpenoids yielded noteworthy compounds, such as the sesquiterpene lactones parthenolide and lactopicrin as well as acetophenone sesquiterpenes and sphaerodiene type diterpenoids, respectively. In addition, pronounced effects were observed for the macrolide neurymenolide A and three flavonol dicoumaroylrhamnosides.

## 1. Introduction

Due to the increasing age of the population, especially in industrialized nations, hospital or nursing home-acquired infections are becoming a major problem (Hasanpour et al., 2023). Antimicrobial resistance is prognosed to cause 10 million annual deaths by the year 2050, rendering it the second leading cause of death after cardiovascular diseases (Nandhini et al., 2022). Many of these infections are associated with methicillin-resistant *Staphylococcus aureus* (MRSA), which has a mortality rate of around 14% in patients with drug-resistant infections in the US (CDC, 2013). However, the prevalence of healthcare-associated MRSA varies for different countries, with, e.g., Portugal (58.4%), Pakistan (52%), India (46%), China (45%), and Norway (38.9%) showing high ratios, while other countries such as Mexico (19.1%), Australia (15.1%), and Germany (4.6%) are much less affected (Shoaib et al., 2022). Even though the first observation of resistance dates back to the 1960s (Jevons, 1961), MRSA is still a threat to health-care systems, evolving more and more resistance mechanisms against a broad spectrum of antibiotics. Apart from community-associated MRSA strains, livestock-associated strains also serve as a reservoir for resistance (Cuny et al., 2013). In 2017, MRSA was classified as a “high priority pathogen” by the world health organization (WHO, 2017), stating the urgent need to develop new treatments against this bacterium, i.e., new substances with antibiotic or antibiofilm activity.

MRSA causes different kinds of infections including bacteremia, skin and soft-tissue infections, infective endocarditis, osteoarticular infections, pleuropulmonary infections, and many more (Tong et al., 2015). This often leads to complications or prolongation of clinical treatments. In the European Union, MRSA causes the second most infections of any drug-resistant pathogen, only surpassed by the cephalosporin-resistant *Escherichia coli* (ECDC, 2021). The treatment includes antibiotics of the so-called drugs of last resort (DoLR) like vancomycin, daptomycin, linezolid, and tigecycline (Choo and Chambers, 2016; Subramani et al., 2017). Alarmingly, strains with resistance to one of these antibiotics have already been detected (Layer et al., 2021).

An overriding cause for the development of microbial resistance is the misuse of antibiotics (Abreu et al., 2012; Peacock and Paterson, 2015; Rossiter et al., 2017). Most resistance mechanisms affect the uptake and efflux of the antibiotic, alter the target structure, modify the antibiotic, or even produce enzymes to inactivate the antibiotic (Peacock and Paterson, 2015; Rossiter et al., 2017). If a strain adopts a resistance the respective gene can be passed along through division or horizontal gene transfer (Peacock and Paterson, 2015; Álvarez-Martínez et al., 2020). Because most of the available antibiotics against MRSA target the cell wall biosynthesis, new lead compounds with other bacteriostatic or bactericidal mechanisms are urgently needed (Choo and Chambers, 2016).

In the past, several natural products have led to effective antibiotics, such as penicillin and vancomycin, with, however, a greater focus on fungal or bacteria-derived compounds (Katz and Baltz, 2016; Wright, 2017; Porras et al., 2021). Nevertheless, plants are a rich source of diverse lead compounds, such as alkaloids, terpenoids, quinones, or polyphenols. These substances protect organisms against harmful bacteria, fungi, and insects, thereby offering greater structural variety than standard small molecule libraries (Subramani et al., 2017; Zaynab et al., 2018; Atanasov et al., 2021). Considering the fact that no new antibiotic class has been approved by the FDA since the late 1980s, the broad diversity of specialized plant metabolites may contribute to the development of future antimicrobials (Durand et al., 2019). The traditional use of medicinal plant species against numerous kinds of infectious diseases is a good starting point for the exploration of new antibiotic lead structures (Anand et al., 2019; Porras et al., 2021). Some plant extracts are already used for the treatment of skin infections caused by MRSA, such as the essential oil from the tea tree *Melaleuca alternifolia* (Myrtaceae) (Nandhini et al., 2022). In addition, not only the pure antibiotic effect is of interest, but also potential additive or synergistic effects with existing antibiotics might be an approach against adapted resistances (Sadeer and Mahomoodally, 2021).

In the following, 223 natural products from various plant species are discussed with regard to their structure and antibacterial effects against 169 different MRSA strains (Supplementary Table S1) as well as for their synergistic effects with a range of different antibiotics.

## 2. Methods

A literature search was carried out using the Web of Science citation index, including all publications published until the end of 2022. The search terms “MRSA” and “natural products” were used yielding 833 results. These were reduced to the field of plant science resulting in 148 hits. Also, other sources were collected, which were not found by database search with the previous terms, yielding a total of 223 plant natural products with reported anti-MRSA activity. The compounds were sorted by compound class, thereby taking compound names and configurations “as is” from the original publications. Plants species names were checked using the “World Flora Online” and eventually changed into the accepted taxa. MIC/IC_50_ values are given in μg/mL using the number of digits given in the original publications. Values found μmol/mL were converted to μg/mL. Values above 100 μg/mL were considered inactive and thus excluded from the review. Activities above 50 μg/mL were referred to as low, MIC and IC_50_ values from 10 to 50 μg/mL as moderate and values below 10 μg/mL as high. For synergistic effects factorial inhibitory concentration index (FICI) values above 2 indicate antagonism, values of 2 to 1 indifference, values of 0.5 to 1 additive effects, and values below 0.5 synergism. The values given in μg/mL represent the MIC of the combination.

## 3. Results and discussion

### 3.1. Alkaloids

The first compound class to be discussed in this review is the class of alkaloids, which are a rich source for analgetic or cytostatic drugs, but are rather rare in the field of antibiotics. However, they acted as lead substances in the development of quinolones, thus remaining important scaffolds in drug development (Cushnie et al., 2014).

**Figure 1:**
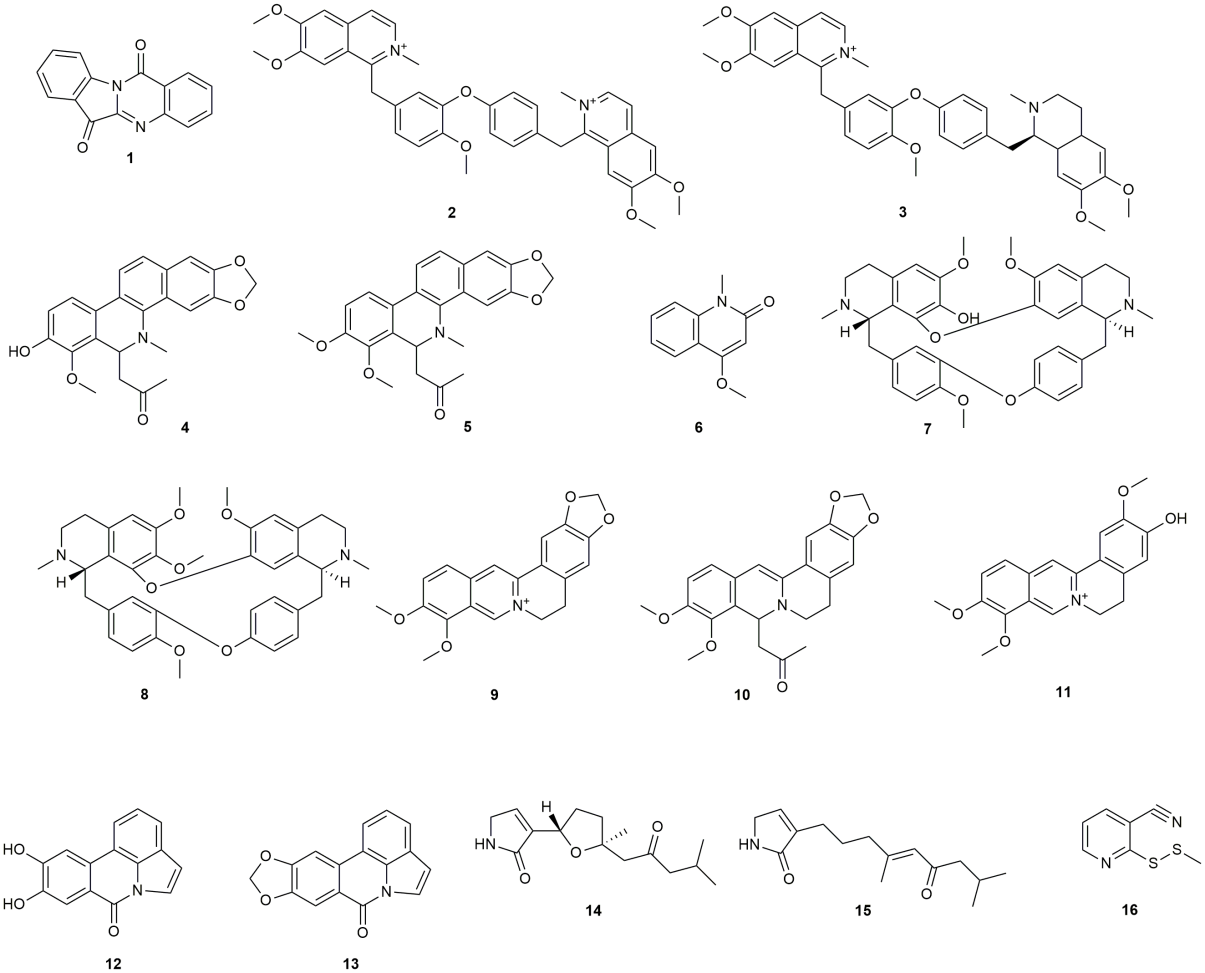
Chemical structures of alkaloids with reported anti-MRSA activity.

Our literature search revealed most reports for isoquinolines-type alkaloids with a total of eight compounds. Additionally, two lycorine derivatives, two pyrolin-2-on derivatives, two phenanthridine-type alkaloids, one quinolone, and one pyridine-derivative were reported (Figure 1).

*Couroupita guianensis*, which is used in traditional South American medicine, yielded **1**, a compound with a low MIC value but also low toxicity (Costa et al., 2017). Liu et al. (2021) extracted two isoquinolines (**2** and **3**) from *Doryphora aromatica* leaves, with activities ranging from 9.9 to 39.6 μg/mL (**2**) and from 19.7 to 39.4 μg/mL (**3**), respectively. Both compounds were first tested against methicillin-sensitive *Staphylococcus aureus* and showed higher activity against the resistant clinical isolates. Another two isoquinolines (**4** and **5**) were isolated from the roots of *Zanthoxylum nitidum* by Zeng et al. (2022). Compound **4** showed moderate and compound **5** showed low activity against MRSA, but both exhibited pronounced synergistic effects with ampicillin, which were attributed to the inhibition of the bacterial efflux pump. Compound **4** was, furthermore, found to successfully disturb the bacterial biofilm. Compound **6**, a quinolone derivative was isolated from *Zanthoxylum schreberi*, formerly named *Zanthoxylum monophylum*, and displayed an IC_50_ value of 1.5 μg/mL, which was rather low compared to the positive control ciprofloxacin (IC_50_ of 0.06 μg/mL) (Rodríguez-Guzmán et al., 2010). Zuo et al. (2011) isolated two bisbenzylisoquinoline alkaloids (**7** and **8**) from *Stephania tetrandra*, a traditional drug in Chinese medicine, with both compounds showing low activity. Yu et al. (2005) and Zuo et al. (2012) both studied compound **9**, an isoquinoline alkaloid from *Coptis chinensis* against different clinical isolates resulting in low to moderate antibacterial effects. However, the compound was found to prevent the bacterium from adhesion and invasion into human gingival fibroblasts, thus also affecting the virulence of MRSA. Additionally, Zuo et al. (2012) isolated **10**, from *Coptis chinensis*, showing a slightly higher activity than **9**. Moreover, **9** and **10** showed synergistic effects with different antibiotics. Also here, the synergistic effect was attributed to the inhibition of the bacterial efflux pump. Yu et al. (2019) tested **11** against an MRSA strain that overexpressed the NorA multidrug efflux pump, thus giving inferences for many wildtype-MRSA strains. While **11** was only slightly active when tested alone, a combination with norfloxacin or reserpine led to an eightfold increase in the antibiotic activity. Therefore, **11** was assumed an efflux pump inhibitor and suggested to be further investigated. The two lycorine derivatives **12** and **13** were isolated from *Crinum ornatum*, formerly referred to as *Crinum distichum*, by Koagne et al. (2018) and showed moderate activity. Two sesquiterpene alkaloids (**14** and **15**) were isolated from the semi-mangrove plant *Myoporum bontioides* (Dong et al., 2018). Both compounds showed high activity (6.5 μg/mL) against the methicillin-resistant but vancomycin-sensitive *S. aureus* strain. Even more pronounced was the effect of **16**, a sulfur-containing pyridine derivative isolated from *Allium stipitatum* (Karunanidhi et al., 2019). The authors additionally performed a time-to-kill assay and observed bactericidal activity after 2 h. Pyridine or thiopyridine-based compounds were reported to have promising antibacterial activity before (Karunanidhi et al., 2019). In addition, compound **16** bears a carbonitrile group, which is also associated with strong antibacterial activity.

Most of the discussed alkaloids exhibit only low to moderate activities against MRSA, with only a few exceptions, such as compounds **14–16**. However, some compounds show auspicious potential as synergistic agents for existing antibiotics through their ability to inhibit the bacterial efflux pump.

### 3.2. Terpenoids

#### 3.2.1. Monoterpenoids and sesquiterpenoids

**Figure 2:**
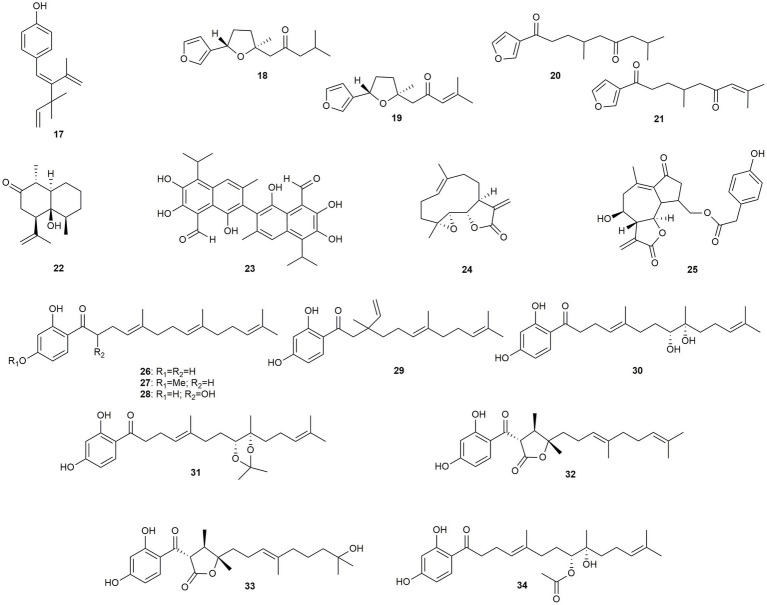
Chemical structures of mono- and sesquiterpenoids with reported anti-MRSA activity.

This section discusses one monoterpenoid and 17 sesquiterpenoids (Figure 2), with the latter compound class being known for some potent antimicrobials, such as artemisinin from *Artemisia annua* or santonin from *Artemisia cina* (Li et al., 2022).

The only monoterpene in this review, nodosol (**17**), was isolated from the marine angiosperm *Cymodocea nodosa* (Kontiza et al., 2008). It showed a MIC value of 16 μg/mL against strains with different resistance mechanisms. Interestingly, the compound showed higher activity than the reference antibiotics against three drug resistant strains. Four furyl bearing sesquiterpenes were isolated from *Myoporum bontioides* (**18–21**), of which two compounds display an additional dihydrofuran feature (**18** and **19**) (Dong et al., 2018). Interestingly, both compounds exhibited twice the activity of their ring-open counterparts (**20** and **21**). Compound **22** was isolated from *Mentha pulegium* and exhibited moderate activity with an IC_50_ value of 8.5 μg/mL (Ibrahim, 2013). The dimeric sesquiterpenoid gossypol (**23**), which is also known from the cotton plant, was isolated from *Thespesia garckeana* by Masila et al. (2015) and showed an IC_50_ of 4.66 μg/mL. Häkkinen et al. (2021) tested the germacranolide lactones parthenolide (**24**, from *Tanacetum parthenium*) and lactucopicrin (**25**, from *Cichorium intybus*) against a β-lactamase possessing strain, with pronounced effects (MIC value of 0.16 μg/mL). *Ferula feruloides* was the source for nine acetophenone sesquiterpenoids (**26**–**34**), which all exhibited activity against different MRSA strains (Sun et al., 2019). **26** (MIC: 1 to 32 μg/mL) showed the highest effect against a strain that expresses a tetK efflux pump and against an epidemic strain. Its *para*-methyl ether **27**, in contrast, was inactive against four out of five strains, indicating the importance of the *para*-hydroxy group for the anti-MRSA activity. This is even more clear when looking at the additional seven acetophenone sesquiterpenes, which (apart from **30**) show pronounced growth inhibition. The number of different compounds in this class allows some further conclusions. Hydroxylation in the α-position of the side chain leads to an increase in activity against almost all strains (**28**, MIC: 1–4 μg/mL), whereas dihydroxylation in position ω-6 and ω-7 results in significantly lower effects (**30**, MIC: 64 μg/mL). Conversely, the formation of a dioxolane ring in the same position (**31**, MIC: 0.5–16 μg/mL) causes a significant increase in activity, especially for the tetK and the epidemic strain. This increase is much less pronounced when only one of the two hydroxy groups derivatized, as, e.g., for compound **34** (MIC: 16–64 μg/mL). Formation of a cyclic lactone in the α-position leads to MIC values of 2–8 μg/mL (**32**) and 2–16 μg/mL (**33**), respectively, whereas a rearrangement of the farnesyl unit toward a non-linear side chain causes even lower activities (**29**).

#### 3.2.2. Diterpenoids

**Figure 3:**
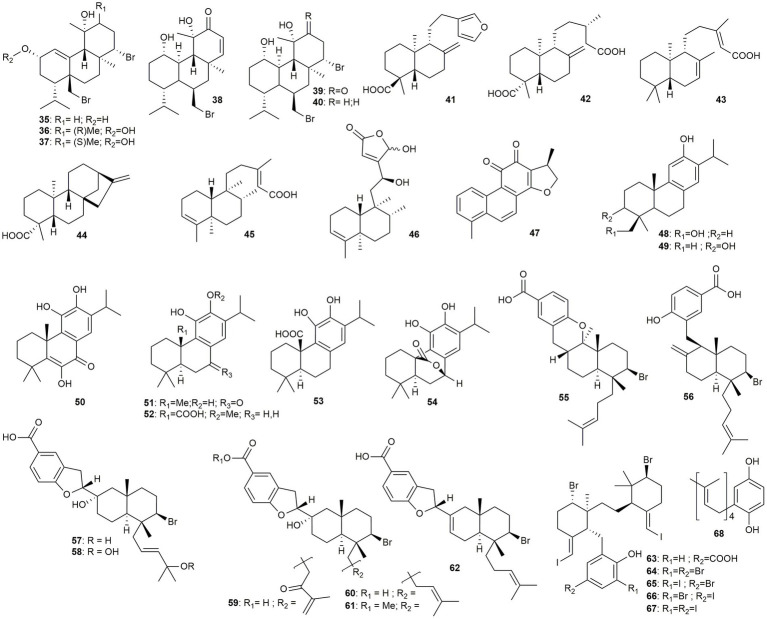
Chemical structures of diterpenoids with reported anti-MRSA activity.

This section comprises 34 diterpenoids of nine different scaffolds, of which most display meroditerpenoids (**55–68**) from the red algae *Callophycus* sp. Additionally, eight abietane type (**47–54**), six sphaerodiene-type **35–40**, three labdane type (**41, 42,** and **43**), two clerodane type (**45** and **46**), and one *ent-*kaurane-type (**44**) diterpenoid will be discussed (Figure 3).

Smyrniotopoulos et al. (2008) isolated six brominated diterpenes (**35–40**) from the red alga *Sphaerococcus coronopifolius*. While compounds **35–37** and **39** were only low to moderately active, compounds **38** (MIC: 0.25 to 1 μg/mL) and **40** (MIC: 1 to 2 μg/mL) showed pronounced effects. The authors suggest that the presence of an α, β-unsaturated ketone at position C-12 may cause the activity, being able to act as a Michael-acceptor. Pfeifer Barbosa et al. (2019) isolated five diterpenoids of three different diterpenoid types (**41–45**) from the oleoresin of *Copaifera reticulata* and tested them for their anti-MRSA activity and their cytotoxicity. Kaurenoic acid (**44**, IC_50_ of 3.4 μg/mL), kolavenic acid (**45**, clerodane-type) (IC_50_ of 3.0 μg/mL), and **43** (labdane-type) (IC_50_ of 2.5 μg/mL) showed the highest activity. Interestingly, the activity was not dependent on the diterpenoid type but increased with the compounds’ lipophilicity. In a follow-up study by Çiçek et al. (2020) compounds **41** and **44** were subjected to semi-synthetic derivatization targeting the exocyclic methylene group as well as the carboxylic acid functionality. Both features were found to be essential for the activity against MRSA. The second clerodane diterpene (**46**) was isolated by Dettweiler et al. (2020) from *Callicarpa americana*. It showed moderate activity (MIC: 16 μg/mL) against a β-lactam resistant strain. Zhao et al. (2021) isolated dihydrotanshinone I (**47**) from *Salvia miltiorrhiza*, which exhibited moderate effects that were proposed to result from disturbance of the cell wall and membrane. Starks et al. (2014) isolated one new and three known abietane-type diterpenoids (**48–51**) with pronounced activities from *Taxodium ascendens*, a species that was reclassified as *Taxodium distichum*. Out of the four compounds, **51** showed lower but broader activity with a MIC value of 4 μg/mL against five different strains. Desaturation and oxygenation in position 5 and 6, respectively, led to comparable activities (**50**, MIC: 1–4 μg/mL) while the exchange of a phenolic hydroxy group in position 11 toward an aliphatic alcohol (in position 3 or 29) led to a significant increase in activity against (at least) two strains (with MIC values of 1–2 μg/mL for compounds **48** and **49**). Oluwatuyi et al. (2004) isolated the three abietane diterpenes, 12-methoxy-*trans*-carnosic acid (**52**), carnosic acid (**53**), and carnosol (**54**), from *Rosmarinus officinalis*. All compounds showed low to moderate activity, with **54** being the most potent compound with a MIC value of 16 μg/mL. Thereby, the lactone bridge seems to be beneficial for the activity compared to a free carboxylic acid. Combinations of **53** and **54** with tetracycline revealed synergistic effects against the TetK-possessing strain XU212. Compound **53**, furthermore, exhibited potent synergistic effects in combination with erythromycin.

Teasdale et al. (2012) isolated six brominated diterpenoids of the sphaerodiene type (**55–60**) from a member of the genus *Callophycus*. Thereby, compounds **59** and **57** were only moderately active, whereas compounds **58** and **55** showed a MIC value of 6.3 μg/mL and compounds **60** and **56** were active with a MIC value of 1.6 μg/mL. The genus *Callophycus* was also the source for eight halogenated meroditerpenoids (**61**–**68**). All compounds showed moderate activity, except compounds **63** (MIC: 1.4 μg/mL), **62** (MIC: 8 μg/mL), and **68** (MIC: 1.8 μg/mL), which exhibited pronounced effects (Lavoie et al., 2017). The structural complexity of this group of diterpenoids derives from the addition of a *para*-hydroxybenzoic acid to the diterpene scaffold either at the methyl group at position 8 (**56**) or at position 9 (**55**). Further condensation of **56** leads to the benzofuran moiety of compounds **57–60,** and **61–62**, respectively. Interestingly, the formation of a dihydropyran ring between the diterpene scaffold and the *para*-hydroxybenzoic acid moiety (as for compound **55**) led to a four-fold lower activity, while the formation of a benzofuran-connected system (**60**) did not alter the antibacterial effect (compared to **56**). Moreover, the formation of a methyl ester (**61**) as well as the oxygenation of the prenyl moiety (as for compounds **57**–**59**) decreased the activity. This effect was less pronounced for the peroxide (**58**), though.

#### 3.2.3. Triterpenoids

**Figure 4:**
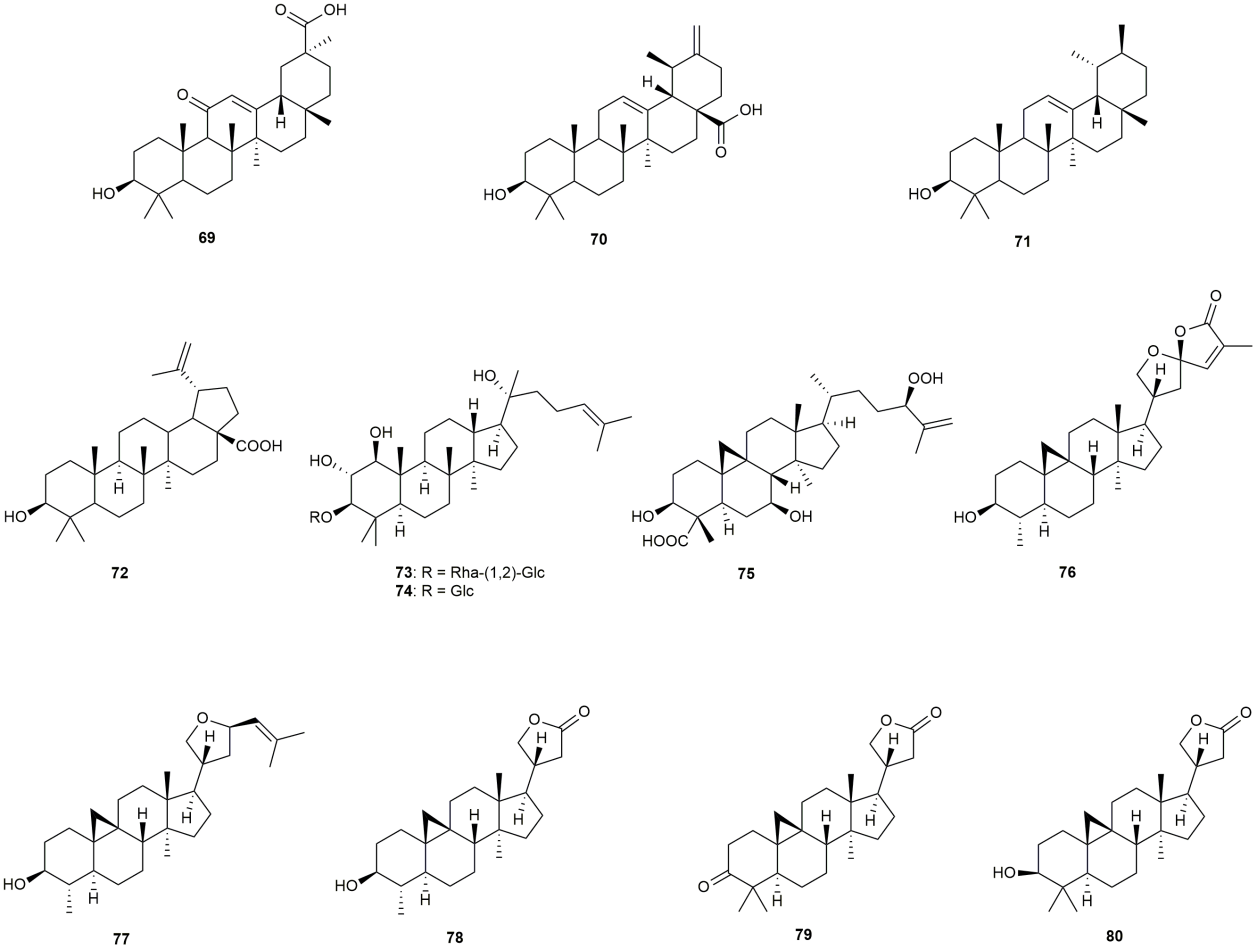
Chemical structures of triterpenoids with reported anti-MRSA activity.

In this section six cycloartane-type triterpenoids, two dammaranes, as well as one lupane, one ursane, one oleane, and one taraxastane will be discussed (Figure 4).

Weaver et al. (2022) isolated 18β-glycyrrhetinic acid (**69**) from the roots of a *Glycyrrhiza* sp. The compound exhibited few effects but lowered MRSA virulence *in vivo*. Micromeric acid (**70**) was isolated by Khin et al. (2021) together with betulinic acid (**72**) from *Rosmarinus officinalis*. Thereby, **72** showed high activity (MIC: 8 μg/mL) against the community-associated strain USA 300, whereas **70** was only moderately active. Chung et al. (2011, 2014) also isolated betulinic acid (**72**) together with α-amyrin (**71**) from *Callicarpa farinosa*, which was later reclassified as *Callicarpa tomentosa*. Both compounds showed moderate activity, with subsequent mechanistic assays revealing promising results against a variety of bacterial transcription mechanisms (Chung et al., 2014). The only two triterpene glycosides (**73** and **74**) were isolated by Garo et al. (2009) from *Oncoba manii*, which is now regrouped into the *Camptostylus* genus, both showing complete growth inhibition at 16 μg/mL. Unfortunately, not enough compound could be isolated for the determination of MIC values. Salam et al. (2021) isolated **75** from the leaves of *Castanea sativa*, showing only moderate anti-MRSA activity. However, further studies showed that the virulence was attenuated by **75**. Five cycloartane triterpenoids were isolated by Wang et al. (2013) from *Aphanamixis grandifolia*, now reclassified as *Aphanamixis polystachya*, of which four compounds (**76–79**) were moderately active (with MIC values ranging from 25 to 50 μg/mL) and one compound (**80**) showed remarkable effects (MIC: 1.57 μg/mL). Thereby not only the hydroxy group in position 3 seems to play an important role for the activity, but also a second methyl group at position 4.

### 3.3. Phenolics

The group of phenolics is divided into seven sections, of which some summarize several smaller compound classes (in terms of the number of compounds with anti-MRSA activity).

Section 3.3.1, i.e., discusses 16 caffeic acid derivatives, of which six are chalcones, three are benzylchromanes, and two are lignans.

**Figure 5:**
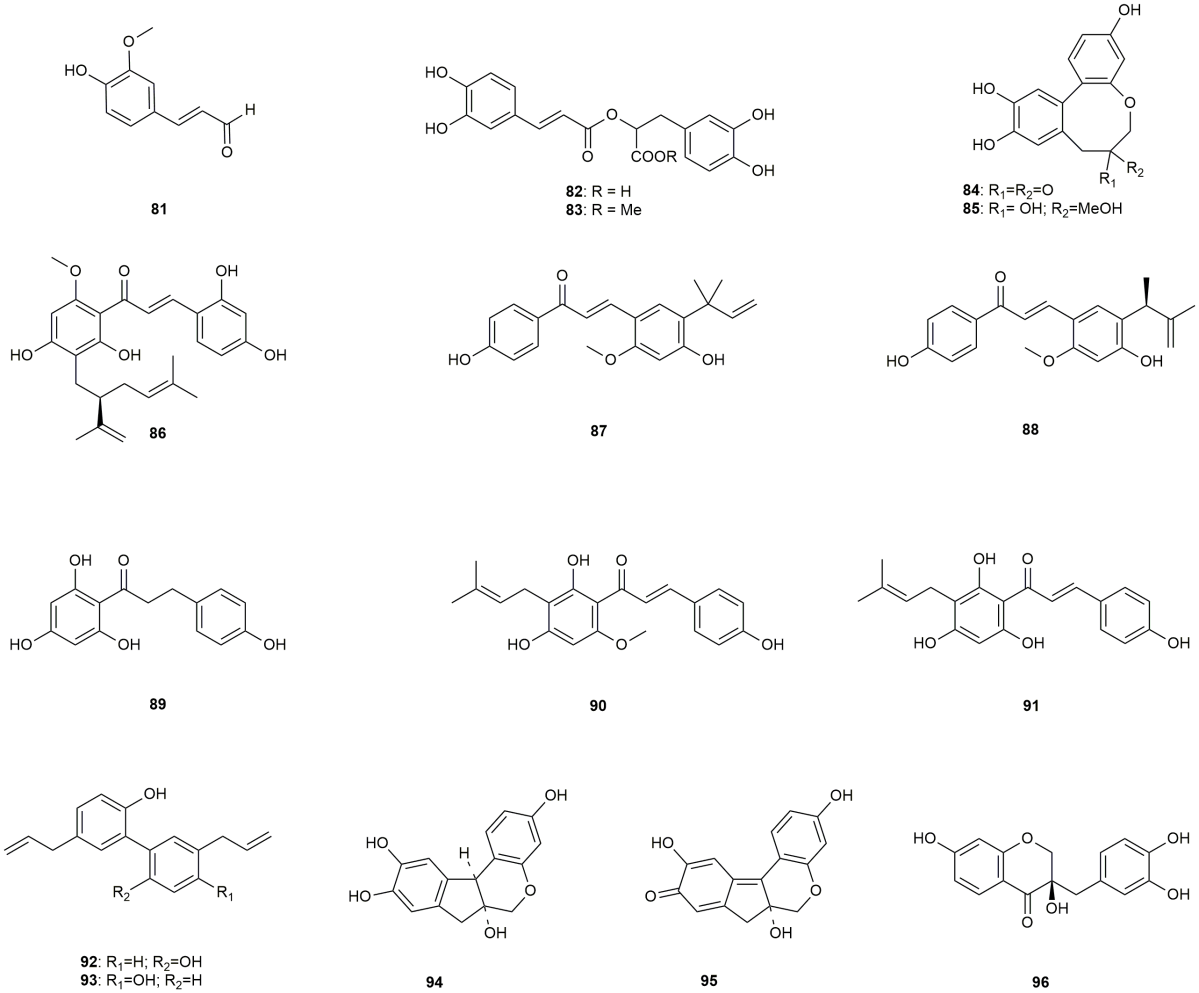
Chemical structures of caffeic acid derivatives with reported anti-MRSA activity.

#### 3.3.1. Caffeic acid derivatives

The root barks of *Cordia gilletii* yielded ferulaldehyd (**81**), which exhibited moderate effects in a study by Okusa et al. (2014). Rosmarinic acid (**82**) and its methyl ester (**83**) were isolated by Sabry et al. (2022) from the stem bark of *Cordia africana.* While **82** was only moderately active (with a MIC value of 31.25 μg/mL), the methylated form (**83**) showed pronounced effects (MIC: 7.81 μg/mL). Zuo et al. (2015) isolated two biphenyl compounds, **84** and **85**, from *Biancaea sappan*, which was previously ascribed to the genus *Caesalpinia*, in a bioactivity-guided isolation procedure. Both compounds showed moderate to low activity but demonstrated synergistic effects in combination with amikacin and gentamycin (Figure 5).

Chan et al. (2012) isolated kuraridin (**86**), a prenylated chalcone, from the traditional Chinese medicial plant *Sophora flavescens* using high-speed counter-current chromatography. The compound showed high activity (MIC: 8 μg/mL) against the efflux pump expressing strains RN4220 (macrolides) and SA-1199B (fluoroquinolones) and against a representative healthcare-associated strain (SA-ST239). Additional checkboard studies found plenty of additive effects in combination with antibiotics, suggesting that the antibacterial action is not directly related to the efflux pump inhibition. Kim et al. (2017) isolated licochalcone A (**87**) and E (**88**) from *Glycyrrhiza inflata*, also known as Chinese licorice. Both compounds showed moderate to high activity (MIC: 10 to 20 μg/mL) against six different strains, even partly surpassing the effect of the positive controls. Lee et al. (2009) tested the dihydrochalcone phloretin (**89**), which was obtained from a compound library. With low toxicity and a MIC value of 16 μg/mL, the authors suggest that the activity against MRSA correlates with the high affinity to the β-ketoacyl-acyl carrier potein synthase III (KASIII), a functional enzyme in the bacterial fatty acid biosynthesis. Bocquet et al. (2019) extracted eight different prenylated phenolic compounds from the female inflorescences of *Humulus lupulus*, which are used for beer brewing. Xanthohumol (**90**) was significantly active with a MIC value of 9.8 μg/mL, while desmethylxanthohumol (**91**) showed only moderate activity against the tested clinical isolates. Moreover, xanthohumol (**90**) displayed synergistic effects in combination with gentamycin, ciprofloxacin, oxacillin, and rifampicin.

Honokiol (**92**) and magnolol (**93**), two lignans from the stem bark of a non-specified member of the genus *Magnolia*, which is used in traditional Chinese and Japanese medicine, were studied by Chiu et al. (2021). Both compounds demonstrated high activity against MRSA (MIC: 10 μg/mL). Further investigations showed that both compounds repressed the expression of mecA, a gene that is important for the β-lactam resistance of *S. aureus*. Additionally, both compounds decreased the biofilm formation in a sub-MIC concentration, which may result from repressing biofilm formation related genes.

Zuo et al. (2014) isolated three 3-benzylchroman derivatives (**94–96**) from the heartwood of the Chinese drug *Caesalpinia sappan* (now *Biancaea sappan*). The compounds showed low to moderate activity with MIC values ranging from 16 to 64 μg/mL. Further studies revealed high synergistic effects of brazilin (**94**) with gentamycin, etimicin, and streptomycin and of brazilein (**95**) with azithromycin, gentamycin, and ceftazidime, repectively.

#### 3.3.2. Flavonoids

**Figure 6:**
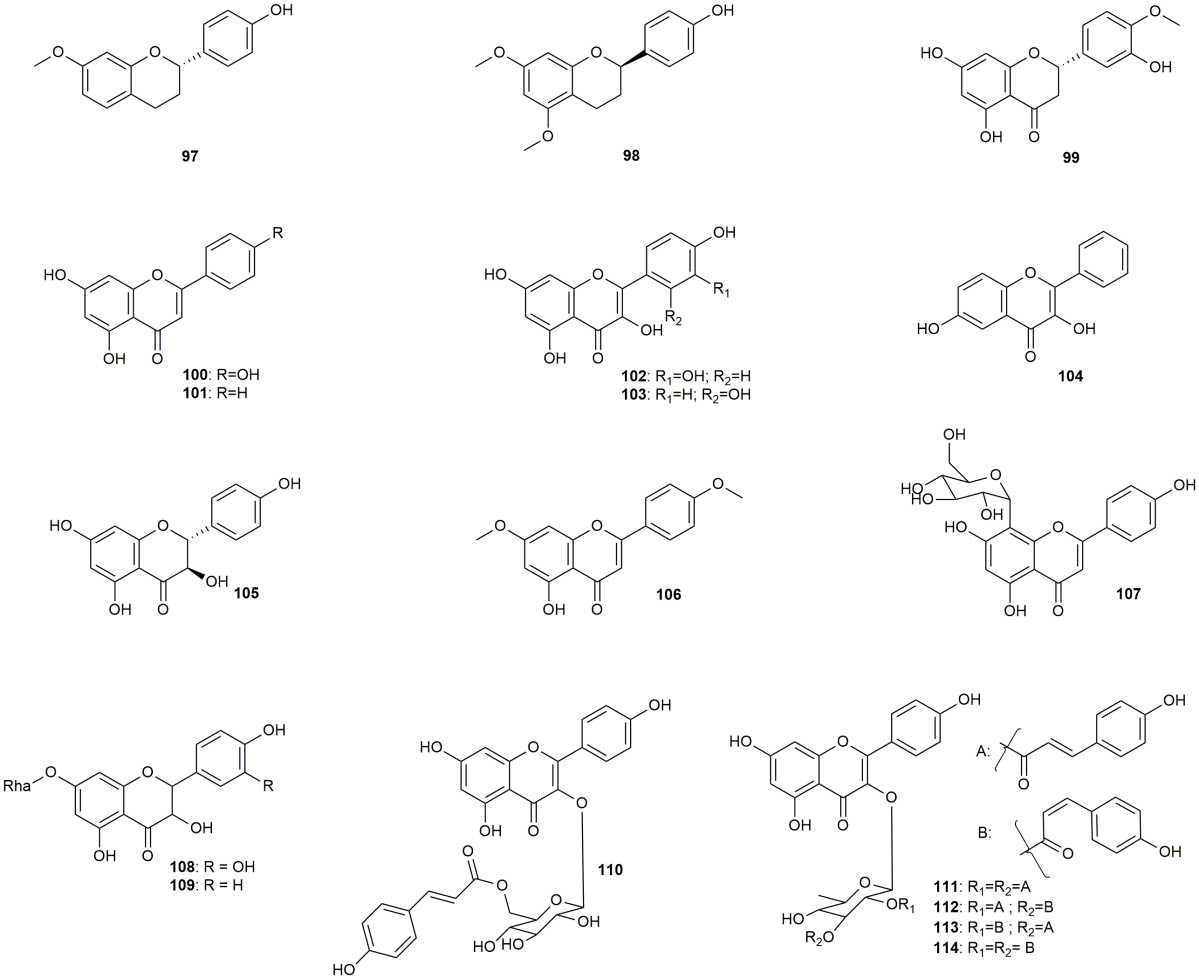
Chemical structures of flavonoids with reported anti-MRSA activity.

The present section discusses eight flavonols, four flavones, three flavanones, two flavans, and one flavanone (Figure 6). Prenylated flavonoids will be dealt with in the subsequent section (3.3.3).

Koagne et al. (2018) isolated **97** and **98** together with two alkaloids from *Crinum distichum*, which was reclassified as *Crinum ornatum*. Both compounds showed moderate activity against a not further specified MRSA strain (Koagne et al., 2018).

Alhadrami et al. (2020) performed a structure-based inverse virtual screening, discovering the penicillin-binding protein 2a (PBP2a) as a potential target for their flavonoid database. PBP2a mediates the antibacterial and antibiotic-synergistic effect of the tested compounds. Subsequently, 23 different flavonoids were investigated, of which quercetin (**102**, MIC: 62.5 μg/mL), apigenin (**100**, MIC: 31.25 μg/mL), chrysin (**101**, MIC: 15.62 μg/mL), and hesperetin (**99,** MIC: 31.25 μg/mL) showed the highest activity against the methicillin-resistant strain ATCC 33591. Because the activity negatively correlated with the number of free hydroxy groups the authors concluded that a lower polarity is beneficial for the positive effects. Pang et al. (2022) isolated morin (**103**) from *Morus alba* with a low activity (100 μg/mL). Lee et al. (2009) performed a receptor-oriented pharmacophore-based *in silico* screening for new inhibitors of the bacterial fatty acid synthase (FAS), revealing 3,6-dihydroxyflavone (**104**) as a potent compound against MRSA (with a MIC value of 16 μg/mL). *Commiphora pedunculata*, a traditionally used medicinal plant species common in Africa, Arabia, and the Indian subcontinent, is the source of dihydrokaempferol (**105**), which displayed moderate activity against a clinical isolate (Tajuddeen et al., 2014). Oluwatuyi et al. (2004) investigated **106** from *Rosmarinus officinalis* against three different drug-resistant strains. Thereby, the highest activity (16 μg/mL) was found against the tetracycline efflux pump possessing strain.

Nzogong et al. (2018) isolated **107** and **110**, two low- to moderately active glycosylated flavonoids from *Dissotis senegambiensis*, which was regrouped into the genus *Antherotoma*, along with four triterpenoids and five tannins. Two flavanonol rhamnosides (**108** and **109**) were isolated by An et al. (2011) from *Hypericum japonicum* and tested against 10 methicillin-resistant and one methicillin-sensitive strain. While **108** was affecting all tested strains, **109** was only inhibiting three strains. However, MIC values of both compounds were only at a moderate to low level (ranging from 32 to 64 μg/mL). The glycosylated flavonoids with the highest activity were isolated from the leaves of *Platanus occidentalis*, the American sycamore (**111**–**114**) (Ibrahim et al., 2009). All four compounds display kaempferol 3-O-dicoumaroylrhamnosides, with different geometric isomerism for the coumaroyl moieties. Thereby, the derivative bearing two *trans*-coumaroyl moieties (**111**) exhibited the lowest activity (MIC value of 10 μg/mL), while two *cis*-coumaroyl moieties (**114**) led to a MIC value of 0.6 μg/mL and thus to a very pronounced effect. Also, compounds bearing one *cis*- and one *trans-* moiety each were still inhibiting at a very high level, with MIC values of 1.7 μg/mL (**112**) and 1.3 mg/mL (**113**), respectively. The isolated compounds were additionally tested against different microorganisms, however, with no observed effects. The position and number of the coumaroyl moiety also seem to have an influence on the activity, considering the low activity of compound **110**. The authors, moreover, suggest that the hydroxy groups at positions 5, 7, and 4′ were important for the anti-MRSA activity (Ibrahim et al., 2009).

The class of (non-prenylated) flavonoids displays mostly low to moderately active constituents. However, for three dicoumaroylrhamnosides pronounced effects have been demonstrated.

#### 3.3.3. Prenylated flavonoids

**Figure 7:**
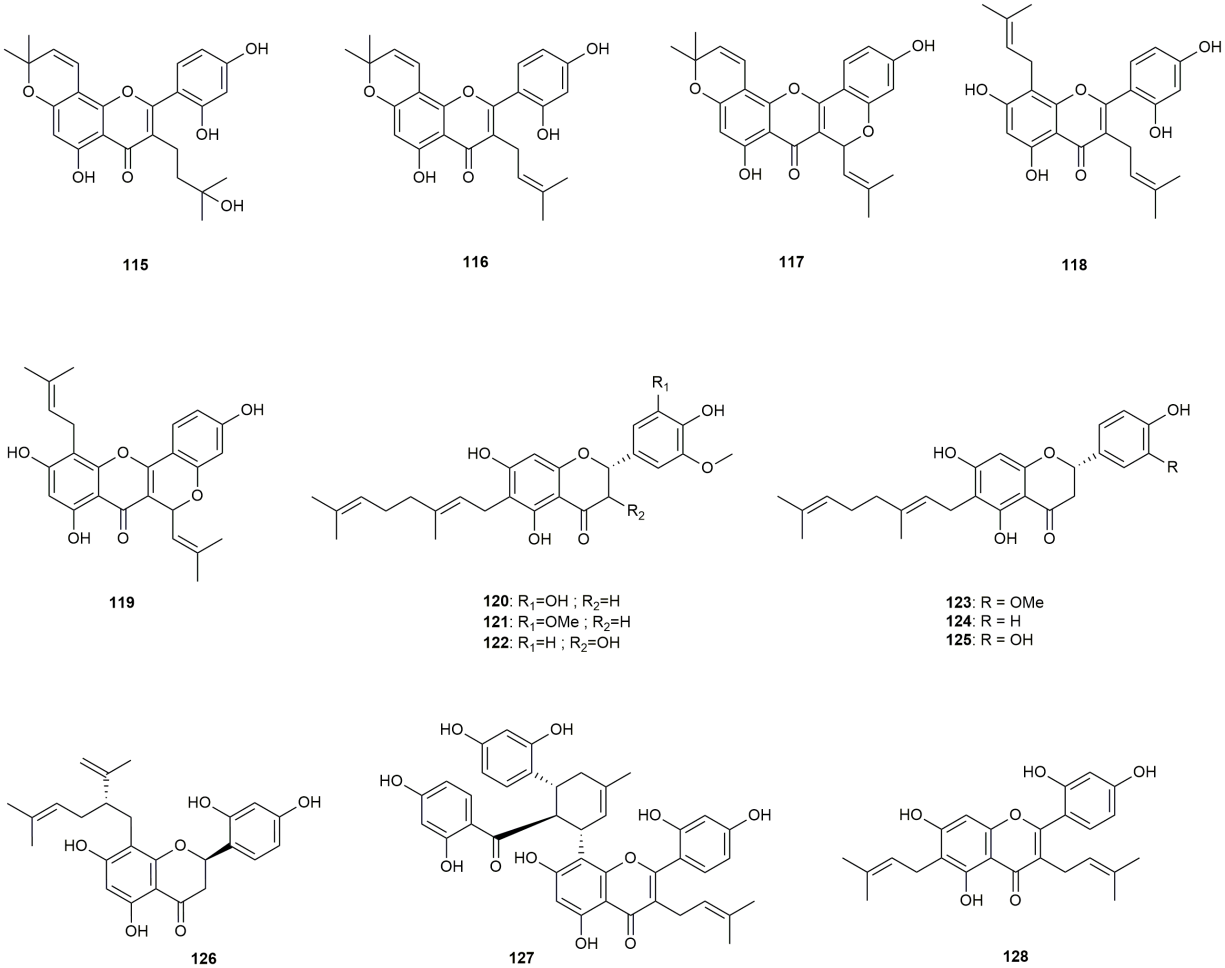
Chemical structures of prenylated flavonoids with reported anti-MRSA activity.

The 14 compounds discussed in this section comprise seven flavones, six flavanones, and one flavanonol-type flavonoid (Figure 7). In addition, there is a broad variety of prenylation pattern, such as mono-, or di-prenylated flavonoids as well as ring or chain prenylated compounds.

Pang et al. (2022) isolated five prenylated flavonoids (**115**–**119**), with activities ranging from low MIC values of 100 μg/mL (**115**) and 74.95 μg/mL (**117**) over 25.02 μg/mL (**119**) to pronounced effects of 12.53 μg/mL (**116**) and 3.08 μg/mL (**118**), respectively. Compound **116** was also tested by Aelenei et al. (2020), who determined a MIC value of 6.25 μg/mL and noteworthy synergistic effects (FICI values of 0.13–0.16) with ciprofloxacin, gentamicin, oxacillin, and tetracycline, respectively. The number of similar structures allow several conclusions. First, the hydration of the C-3 prenyl chain almost completely abolishes the antimicrobial effect and second, ring closure between the B- and C-ring also leads to a decrease in activity. Furthermore, chain prenylation in the α-position (C-8) results in a significantly higher activity than ring prenylation.

Navrátilová et al. (2016) studied six C-6-geranylated flavonoids (**120**–**125**) isolated from *Paulownia tomentosa* against six MRSA strains with additional resistance against tetracycline. All compounds showed high to moderate effects, with mimulone (**124**, MIC values ranging from 2 to 16 μg/mL) and 3-O-methyldiplacol (**122**, MIC values of 4–8 μg/mL) exhibiting the most pronounced activity. The latter two compounds were further investigated for their synergistic effect resulting in additive effects with oxacillin against most MRSA strains. The authors suggest that the synergistic effect is caused by the inhibition of PBP2a and bacterial efflux pumps or by interactions with membrane peptidoglycans.

Chan et al. (2012) isolated sophoraflavanone G (**126**) from *Sophora flavescens*. The compound showed high activity in the range of 2 to 4 μg/mL, but was also found cytotoxic at the same concentration. Kuwanon G (**127**) was isolated from *Morus* sp. by Aelenei et al. (2020) and showed a MIC value of 12.5 μg/mL as well as synergistic effects with four different antibiotics. Even more active was cudraflavone C (**128**), which was isolated from *Artocarpus hirsutus* and tested against 12 different MRSA strains (Meenu et al., 2021). The compound affected all strains with a MIC value of 4 μg/mL, induced a reduction of biofilm formation by 30.4% and showed synergistic effects in combination with gentamycin.

#### 3.3.4. Isoflavonoids

**Figure 8:**
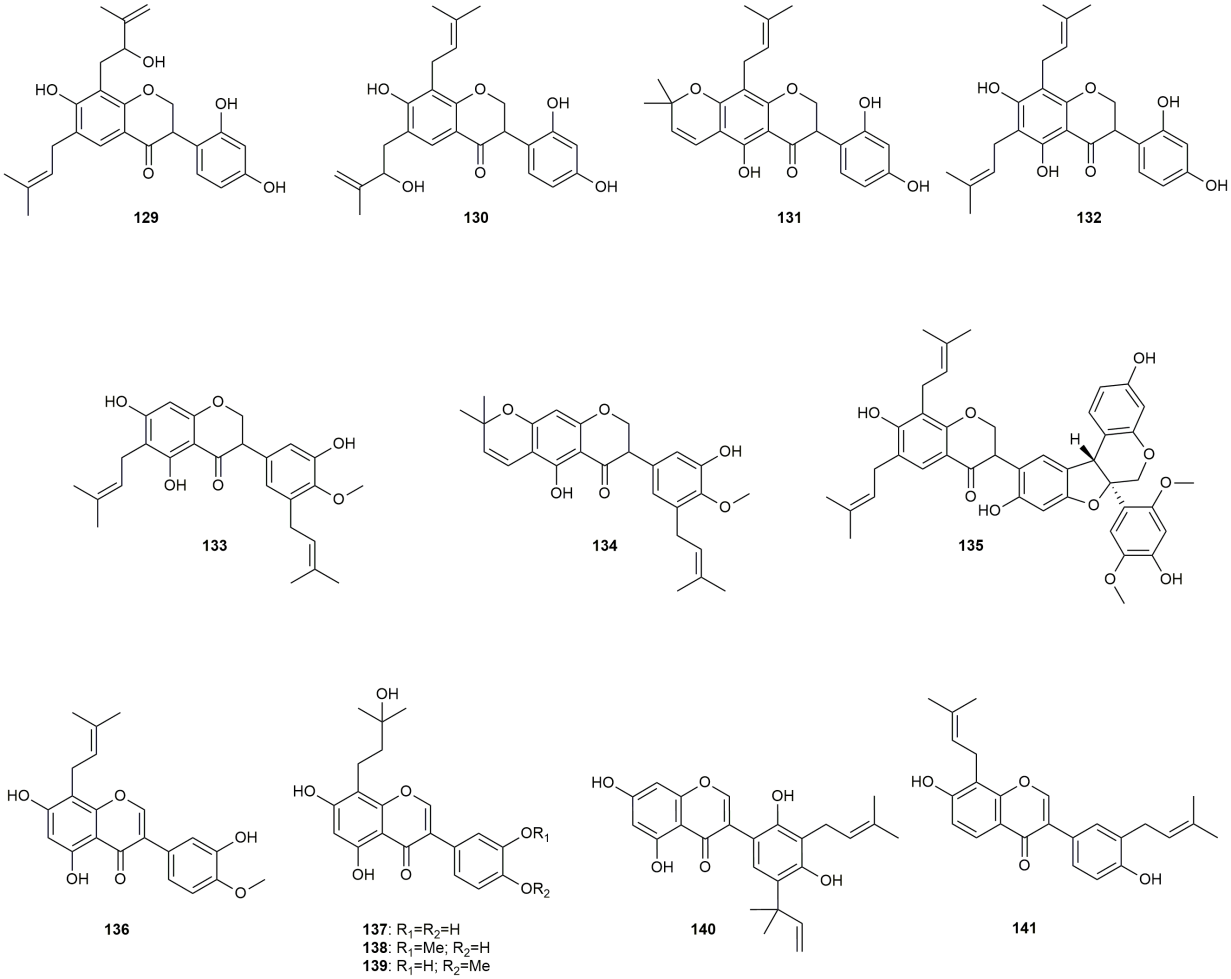
Chemical structures of isoflavonoids with reported anti-MRSA activity.

In addition to the prenylated flavonoids there is a total of 13 isoflavonoids discussed in this review, with seven of them being isoflavanones and six being isoflavones (Figure 8).

Seven isoflavanones with widely ranging activities (**129–135**) were isolated from *Erythrina variegata* and *Erythrina costaricensis* by Tanaka et al. (2009, 2015). While compounds (**129**, **130**, **134**, and **135**) only showed moderate effects against 13 different MRSA strains, promising activities were observed for compounds **131** to **133**. Interestingly, in both cases (**132** vs. **131** and **133** vs. **134**) chain prenylation at position C-6 led to a much higher activity than ring prenylation at position C-6/O-7. Çiçek et al. (2022) isolated four isoflavones with chain prenylation in the α-position (**136–139**) from *Vatairea guianensis*. While vatairenones C**–**E (**137–139**) were only moderately active, compound **136** exhibited pronounced effects with an IC_50_ value of 6.8 μg/mL. Fremontone (**140**), a prenylated isoflavone isolated by Kumarihamy et al. (2022) from *Psorothamnus schottii* showed moderate activity (MIC value of 12.5 μg/mL) as well as synergistic effects with methicillin and no cytotoxic activity. Kwesiga et al. (2020) tested erysubin *F* (**141**), an isoflavone isolated from *Erythrina sacleuxii*, also resulting in moderate activity (MIC value of 15.4 μg/mL). The authors compared the activity of **141** with its non-natural C2-C3 flavone isomer, finding that the isoflavone structure has a minor influence on the activity than the higher lipophilicity caused by two prenyl groups.

#### 3.3.5. Gallic acids, stilbenoids, phenanthrenes, biphenyls, and diarylheptanoids

**Figure 9:**
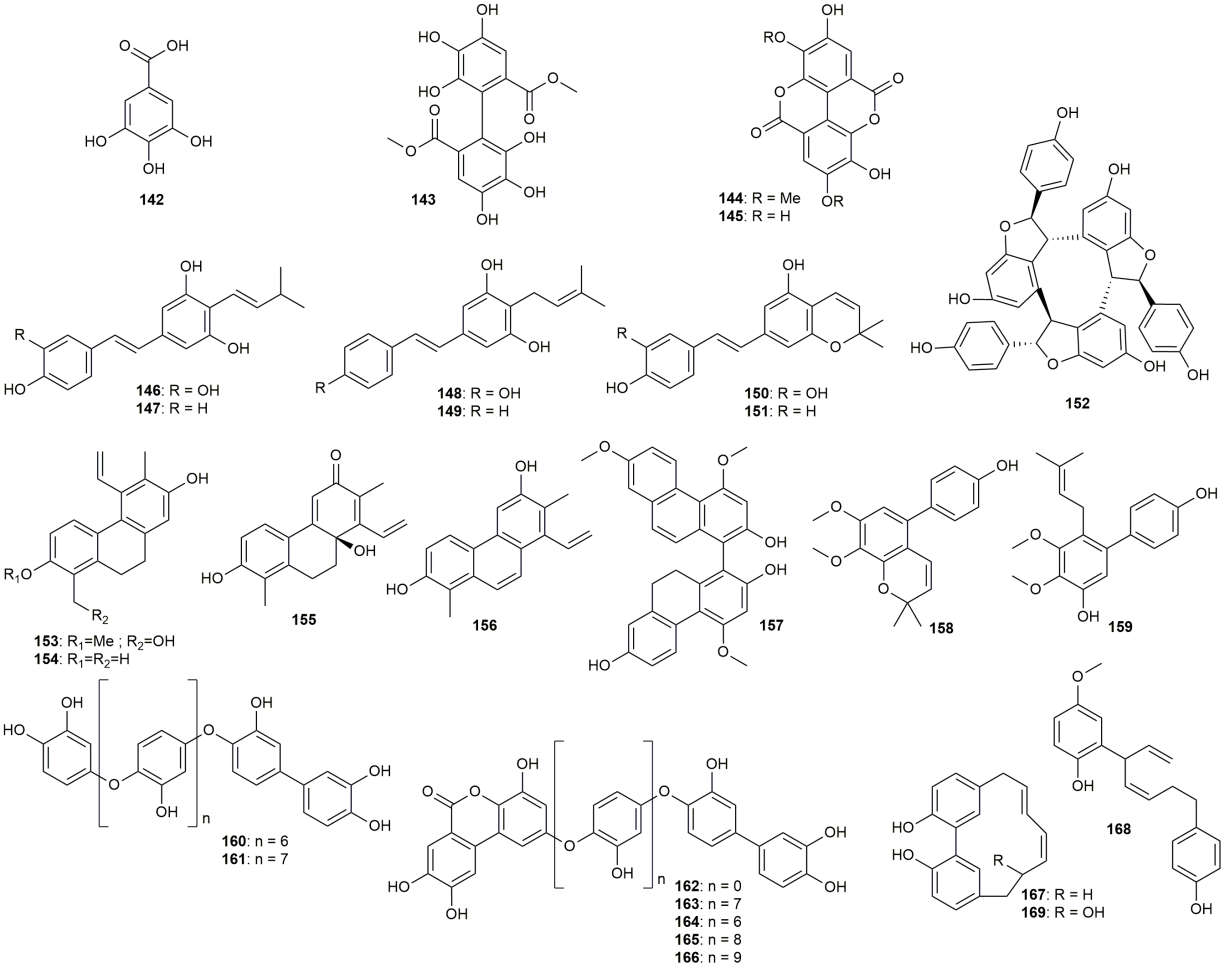
Chemical structures of gallic acids, stilbenoids, phenanthrenes, biphenyls, and diarylheptanoids with reported anti-MRSA activity.

The following section discusses gallic acid and three of its dimers, seven stilbenoids, 14 biphenyl derivatives, and three diarylheptanoids (Figure 9).

Gallic acid (**142**) was isolated by Lakornwong et al. (2018) from the stems of *Rhodamnia dumetorum*, which is traditionally used for its astringent effect. The compound only showed moderate activity, with a MIC value of 16 μg/mL. However, also the three ellagic acid derivatives (**143**–**145**) isolated from *Amphiblemma monticola* only exhibited low to moderate effects (MIC values of 16–64 μg/mL) (Nzogong et al., 2018).

Bruijn et al. (2018) isolated six prenylated stilbenoids (**146**–**151**) from peanut (*Arachis hypogaea*). The antibacterial activity was assessed together with their non-prenylated precursors piceatannol, resveratrol, and pinosylvin. The precursors were found inactive, whereas the tested prenylated stilbenoids showed moderate to low activity (MIC: 12.5 - >50 μg/mL), i.e., arahypin-5 (**151**), the ring-prenylated derivative from resveratrol showing an up to tenfold increase in activity. The highest activity was determined for chiricanine A (**149**) (MIC: 12.5 μg/mL), the only compound with no hydroxy group in the B-ring. Seo et al. (2017) isolated the stilbene trimer α-viniferin (**152**) from the roots of *Carex humilis*, which possessed profound activity against MRSA (MIC: 6.24–12.49 μg/mL). The compound was furthermore investigated in a clinical trial, where it reduced antimicrobial activity of different *Staphylococcus* species (including MRSA) in the nares of 20 Korean females without depleting the normal microbiota (Rahim et al., 2021).

Four phenanthrene derivatives (**153**–**156**) were isolated by Tóth et al. (2016) from *Juncus inflexus*. Antibacterial screenings resulted in juncuenin D (**155**) as the most potent anti-MRSA agent (MIC value of 12.5 μg/mL). Additionally, juncusol (**154**) and dihydrojuncuenin B (**156**) showed a MIC value of 25 μg/mL, whereas jinflexin B (**153**) was almost inactive. Chen et al. (2018) isolated blestriacin (**157**), a dimeric phenanthrene derivative, from the fibrous roots of *Bletilla striata*. The compound showed high activity against the standard strain and two clinical isolates (MIC: 2 μg/mL). To further investigate the mechanism of action of **157**, a growth kinetic assay was performed comparing the compound to antibiotics with known mechanisms. Interestingly, blestriacin (**157**) exhibited a growth kinetic similar to rapidly lytic membrane-active agents like polymyxin and nisin. Therefore, disrupting membrane potential and integrity could be a possible mechanism of action. In addition, two moderately active prenylated biphenyls (**158** and **159**) were isolated from *Garcinia esculenta* by Zheng et al. (2021).

Lavoie et al. (2019) isolated seven oligomeric biphenyls (**160**–**166**) from the green alga *Cladophora socialis*. The highest activity was determined for cladophorol C (**160**) with a MIC value of 1.4 μg/mL. Thereby, those oligomers which were not condensed with protocatechuic acid exhibited higher effects. Moreover, the number of monomers seem to have an impact on the activity, with the most potent compounds containing nine to ten monomers.

*Cymodocea nodosa*, a marine angiosperm, yielded three diarylheptanoids (**167**–**169**) in a study by Kontiza et al. (2008). All of the tested compounds showed weak to moderate activity, with MIC values ranging from 64 μg/mL (**169**) to 32 μg/mL (**167**).

#### 3.3.6. Xanthones and anthranoids

**Figure 10:**
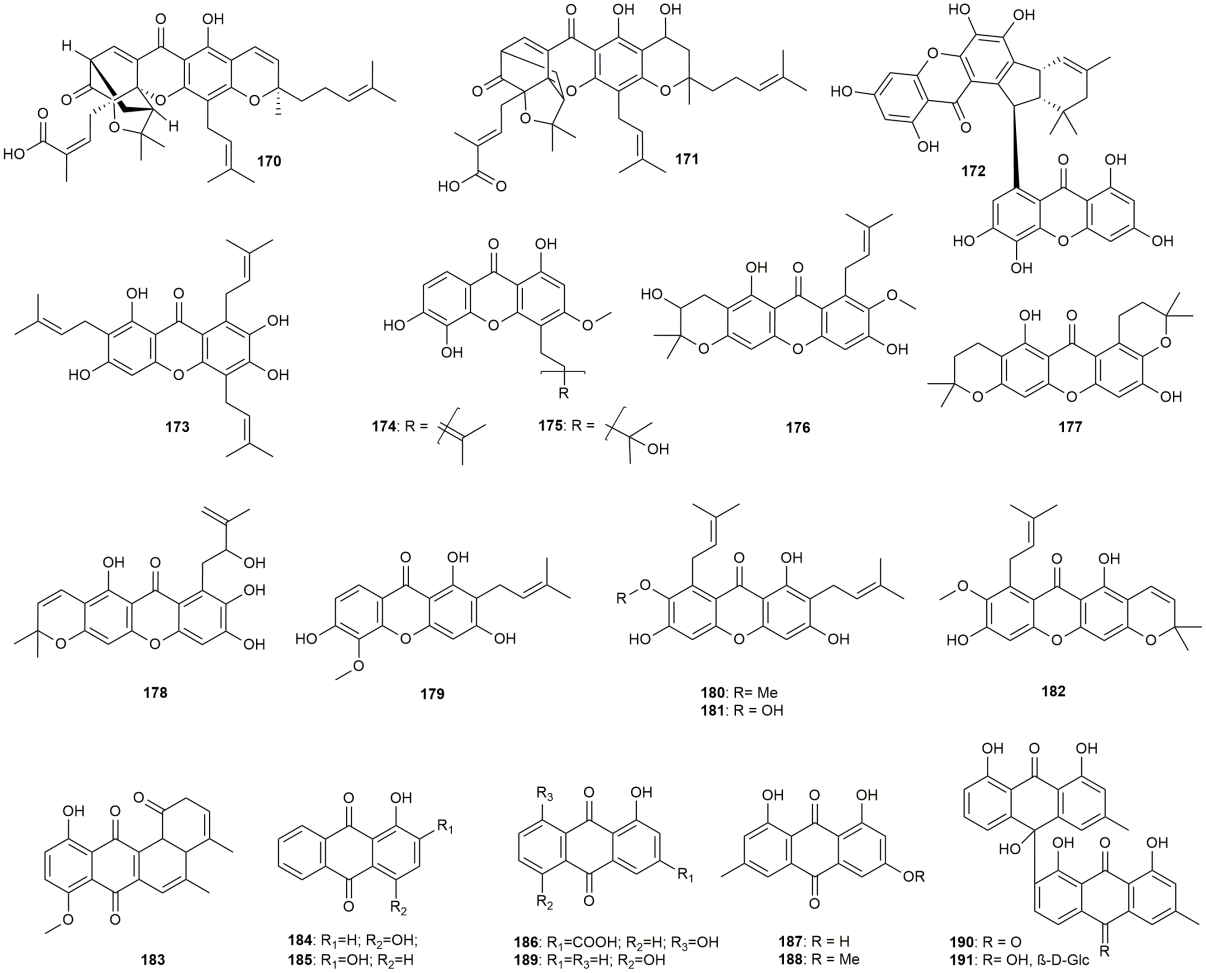
Chemical structures of xanthones and anthranoids with reported anti-MRSA activity.

Twelve prenylated xanthones and one dimer as well as seven anthrachinones and two dimeric anthranoids will be discussed in this section (Figure 10).

Hua et al. (2019) investigated the two prenylated polycyclic xanthone derivatives gambogic and neogambogic acid (**170** and **171**) for their anti-MRSA activity, their cytotoxicity, and their ability to inhibit the adhesion and infection of cells. Both compounds showed pronounced activity against MRSA with a MIC value of 1 μg/mL. One dimeric xanthone (**172**) and five prenylated monomers (**172–177**) were isolated from *Garcinia mangostana*, a plant native to Asia, Australia, tropical, and South Africa (Wang et al., 2018). The dimer garmoxanthone (**172**) as well as compounds garcinol E (**173**) and dulxanthone A (**174**) showed strong activity with MIC values of 3.9 μg/mL. Compounds **175**–**177**, in contrast, only exhibited low to moderate effects (MIC: 31.2–62.4 μg/mL). Another two prenylated xanthones (**178** and **179**) were isolated from *Garcinia esculenta* and showed moderate to high effects (MIC values of 6.25–25 μg/mL) (Zheng et al., 2021). *Garcinia mangostana* is also the source for the three prenylated xanthones α-mangostin (**180**), γ-mangostin (**181**), and 9-hydroxycalabaxanthone (**182**), which all exhibited pronounced effects (Dharmaratne et al., 2013). While the latter compound (**182**) was active with a MIC value of 16.66 μg/mL, α- and γ-mangostin (**180** and **181**) even showed MIC values of 6.25 and 3.13 μg/mL, respectively.

Interestingly, the common feature of the highly active compounds (**173**, **174**, **179**, **180**, and **181**) is the missing ring prenlyation attached to the B-ring of the xanthone scaffold. Additionally, multiple chain prenylation and/or methylation of one of the hydroxy groups contribute to the activity, indicating the lipophilicity of the compounds as an important feature for the anti-MRSA effect.

Phytochemical investigations of *Stereospermum fimbriatum*, a plant that is traditionally used for itchy skin, earache, stomachache, and postpartum treatment yielded the angucycline derivative **183**, which displayed a MIC value of 6.25 μg/mL (Awang et al., 2020). Five anthraquinones (**184–189**) were studied in a *in silico*-based screening for new anti-microbial agents by Alhadrami et al. (2022). Quinizarin (**184**) and alizarin (**185**) showed weak activity, while rhein (**186**) was moderately active. Emodin (**187**), physcion (**188**), and anthrarufin (**189**), however, exhibited pronounced effects with MIC values of 2 μg/mL. Rhein (**186**) was tested also by Joung et al. (2012) with MIC values between 7.8 and 31.25 μg/mL. In another study, emodin (**187**) was isolated from *Rheum palmatum*, and tested by Lee et al. (2010) resulting in MIC values of 1.56–25 μg/mL. Emodin (**187**) furthermore, showed synergistic effects with amoxicillin and oxacillin. One dianthrone (**190**) and its glycoside aestivin (**191**) were isolated from the tubers of *Asphodelus microcarpus* (Ghoneim et al., 2013). Both compounds showed promising IC_50_ values of 9.4 μg/mL (**190**) and 1.4 μg/mL (**191**), respectively. Interestingly, in this study the glycoside and thus the less lipophilic derivative exhibited stronger effects than its aglycone counterpart.

#### 3.3.7. Phloroglucinol derivatives

**Figure 11:**
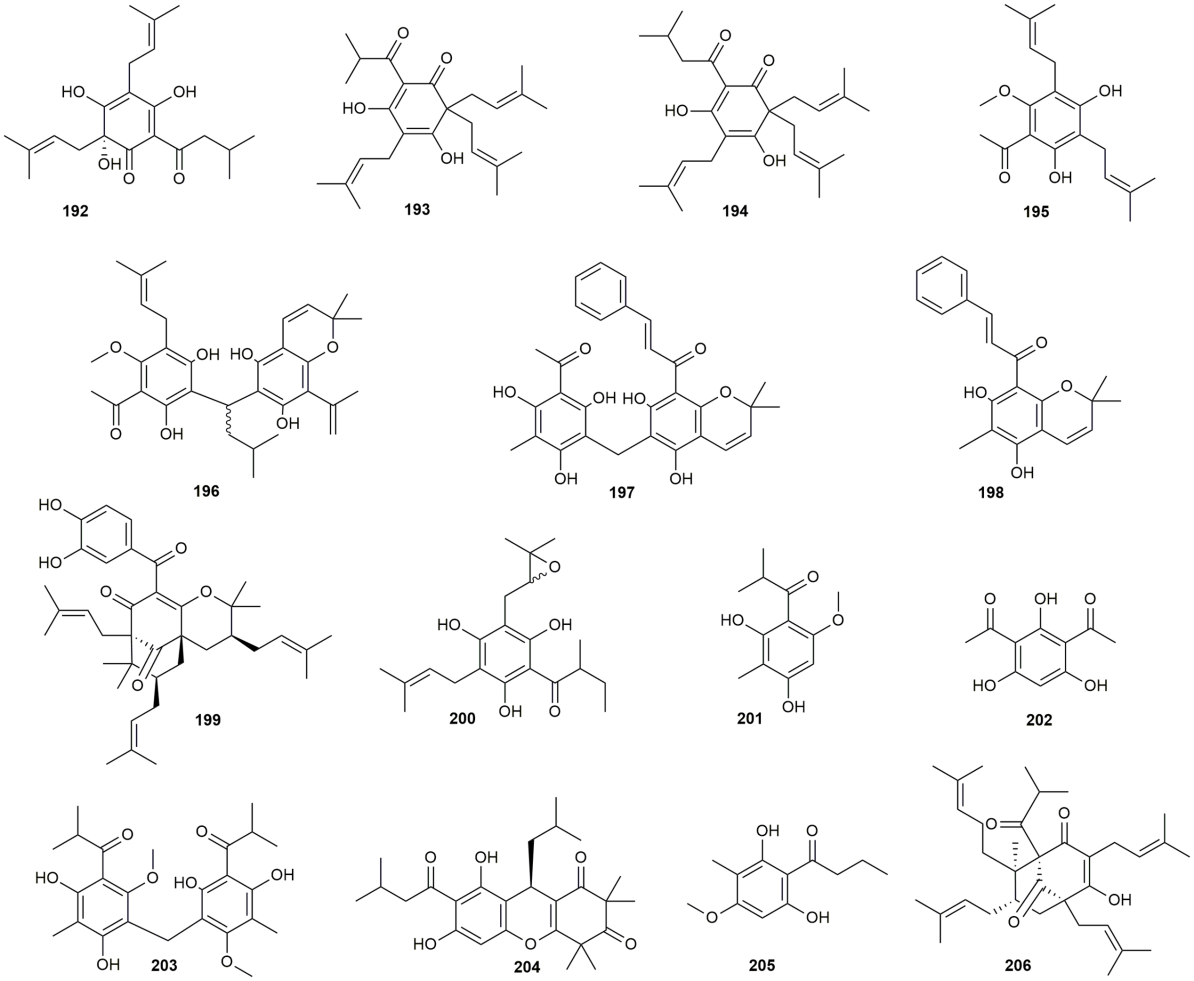
Chemical structures of phloroglucinol derivatives with reported anti-MRSA activity.

In this section, a total of 15 phloroglucinol derivatives will be discussed, some of which belong to the most active compounds in this review (Figure 11).

Bocquet et al. (2019) isolated three acyl phloroglucinols (**192–194**) from hops (*Humulus lupulus*). While humulone (**192**) and colupulone (**193**) showed only weak to moderate activity, lupulone (**194**) exhibited remarkable effects with a MIC value of 0.6–1.2 μg/mL. Wisetsai et al. (2022) extracted two prenylated phloroglucinol derivatives (**195** and **196**) from the fruit of *Acronychia pedunculata*. Also here, one of the two isolates (**195**) was only moderately active, while the dimeric compound **196** showed pronounced effects (MIC value of 8 μg/mL). Another highly active dimer, namely rottlerin (**197**) was isolated from *Mallotus philippensis* (Oyedemi et al., 2016). While the monomer **198** showed only moderate activity, the dimer rottlerin (**197**) demonstrated high activity (MIC: 2–8 μg/mL) against six out of nine different strains. Wang et al. (2018) isolated garcinol (**199**) from *Garcinia mangostana* and determined a MIC value of 15.6 μg/mL against two different strains. With a MIC value of 16 μg/mL, the acyl phloroglucinol **200** isolated from *Hypericum foliosum*, showed comparable activity (Gibbons et al., 2005). Ahmed et al. (2014) studied the antibacterial effect of two monomeric (**201** and **202**) and one dimeric (**203**) phloroglucinol derivative from *Ivesia gordonii*. Thereby, **201** was found moderately active, while 2,4-diacetylphloroglucinol (**202**) exhibited pronounced effects. Even more interesting was the activity of ivesinol (**203**), which with a MIC value of 0.31 μg/mL is one the most promising plant natural products with anti-MRSA activity. Rhodomyrtone (**204**) was purified by Sianglum and Sirmanote (2012) from *Rhodomyrtus tomentosa* and likewise showed promising activity with a MIC value of 0.5 μg/mL. Aspindol (**205**), which was isolated from the fern *Dryopteris fragrans*, was studied for its anti-MRSA and anti-biofilm activity and for its mechanism of action (Hua et al., 2018). The compound demonstrated high activity against 19 clinical isolates with MIC values ranging from 0.5 to 2 μg/mL. Additional RNA-sequencing of the MRSA strain revealed the downregulation of genes involved in the ribosome and amino acid synthesis, iron transport, and β-lactam resistance. The last phloroglucinol derivative in this review, hyperforin (**206**), was isolated from *Hypericum perforatum* and was as well tested for its anti-MRSA and anti-biofilm activity (Schiavone et al., 2013). Also, for hyperforin (**206**) significant antibacterial effects were determined with MIC values of 0.5–1 μg/mL, thus confirming the potential of phloroglucinol derivatives as anti-MRSA lead compounds.

### 3.4. Additional phenolic and non-phenolic compounds

**Figure 12:**
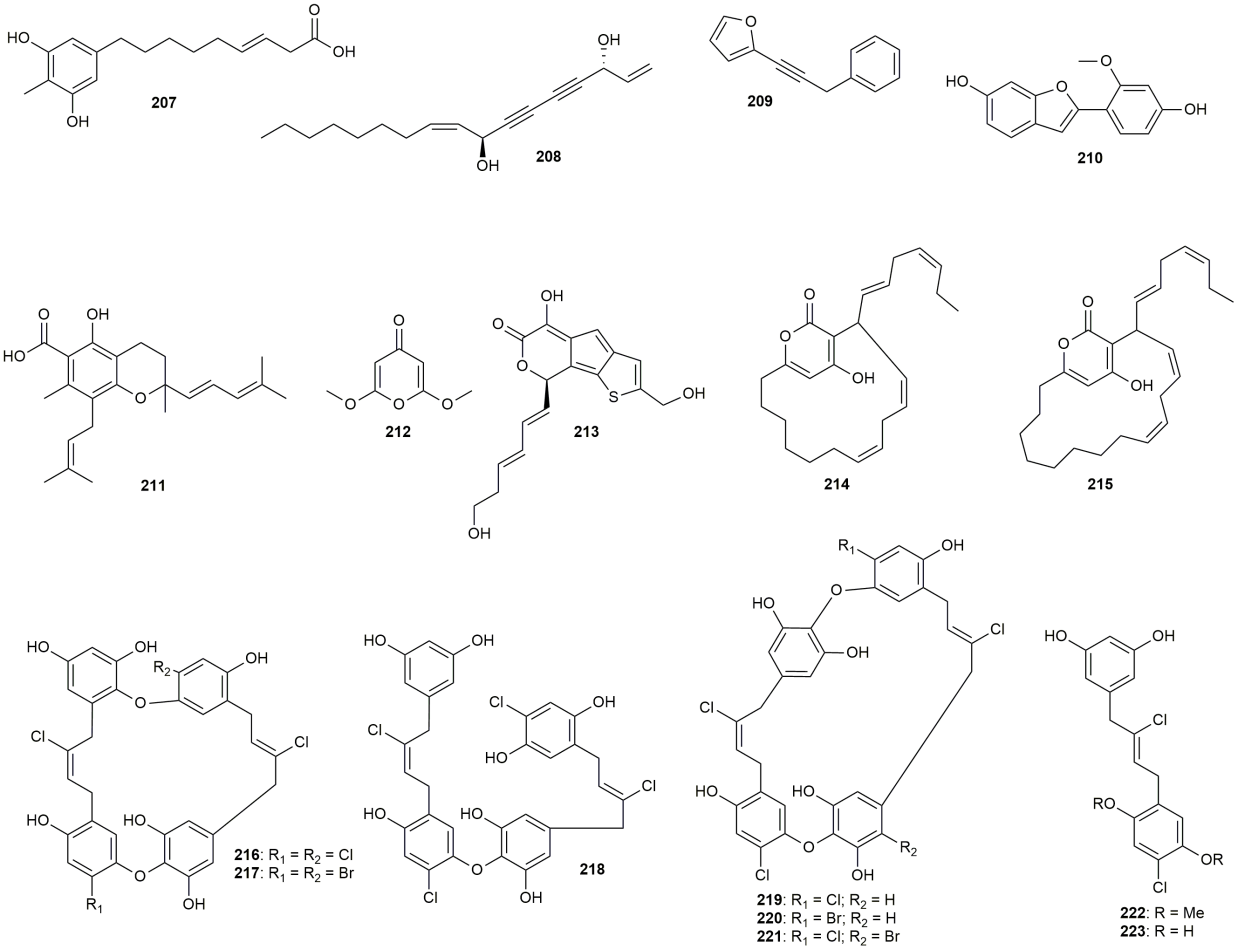
Chemical structures of additional phenolic and non-phenolic compounds with reported anti-MRSA activity.

The last section of this review summarizes natural products, which could not be attributed to the before discussed compound classes and were not enough in number to create respective sections. These compounds comprise one acylresorcinol, one mono- and one polyacetylene, one benzofuran, one prenylated benzopyran, one pyran derivative, one sulfur containing cyclopentapyran, two macrolides, and seven chrysophaentins (Figure 12).

Luís et al. (2013) isolated an acylresorcinol derivative (**207**) from the fruits of *Hakea sericea*, which only showed moderate effects (MIC value of 20 μg/mL) against two strains.

A natural polyacetylene, namely falcarindiol (**208**), was tested by Lechner et al. (2004) after extraction from *Angelica dahurica*. The compound showed high to moderate activity, with MIC values ranging from 8 to 32 μg/mL. The monoacetylene **209** was isolated from the essential oil of *Carlina acaulis* by Herrmann et al. (2011). The compound showed moderate activity, with a MIC value of 15 μg/mL.

Tanaka et al. (2015) isolated a benzofuran derivative (**210**) from *Erythrina variegata*, which, however, only exhibited moderate effects.

Ruiz Mostacero et al. (2021) extracted the prenylated benzopyran **211** from *Peperomia obtusifolia* in a bioassay-guided isolation approach. The compound showed high activity (MIC: 4 μg/mL) against the community-associated strains. Moreover, fluorescence microscopy revealed the ability of **211** to disturb and interact with the bacterial cell wall, which could be due to the amphipathic characteristics of the compound. Also, the pyran derivative **212**, isolated from *Rhodamnia dumetorum*, exhibited pronounced effects with a MIC value of 8 μg/mL (Lakornwong et al., 2018).

The sulfur-containing benzopyran thienocyclopentapyran (**213**) from *Robus hirsutus*, a species now reclassified as *Robus pannosus*, in contrast, exhibited remarkable effects with a MIC of 1 μg/mL (Lin et al., 2021).

The Fijian red alga *Neurymenia fraxinifolia* is the source for the two macrolides neurymenolide A (**214**) and B (**215**). The compounds showed IC_50_ values of 0.77 μg/mL (**214**) and 3.09 μg/mL (**214**), respectively, with the compound having a smaller ring size exhibiting stronger effects (Stout et al., 2009).

Eight chrysophaentins (**216**–**223**), a type of bisdiarylbutene macrocycles, were isolated from the alga *Chrysophaeum taylori* (Keffer et al., 2012). The compounds showed weak to moderate effects (MIC values from 17 to 74 μg/mL) with only one compound demonstrating pronounced activity (chrysophaentin A, **216**, MIC: 4.6 μg/mL). Interestingly, chrysophaentin A (**216**) and D (**217**) only differ by the substitution of two chlorine atoms versus two bromine residues, but show a 15-fold change in activity. Therefore, the lower electron radius of chlorine compared to bromine seems to play a role in the activity, which is also evident for compounds chrysophaentin F (**219**, MIC: 19 μg/mL) and H (**221**, MIC: 17 μg/mL) versus chrysophaentin G (**220**, MIC: 17 μg/mL).

## 4. Current status and future perspectives

In the last 40 years, more than 160 antibacterial drugs were approved by the U.S. Food and Drug Administration, half of them being of natural origin or derived from natural leads (Newman and Cragg, 2020). Still, none of these compounds were isolated from plants though several plant natural products exhibited noteworthy effects at a preclinical stage. In the case of MRSA, the many kinds of infections, e.g., skin and soft tissue infections, infective endocarditis, or pleuropulmonary infections, are certainly hindering when it comes to clinical trials (Tong et al., 2015). Especially, with regard to topical applications the amount of drug material that is needed stands against the limited amounts obtained by isolation procedures. There have been significant technical approaches toward the discovery of potential drug candidates, e.g., molecular networking and machine learning approaches (Fox Ramos et al., 2019; Vamathevan et al., 2019), their fast and efficient isolation, e.g., counter-current chromatography or centrifugal partition chromatography (Ito and Bowman, 1970; Murayama et al., 1982), and their final structure elucidation at even sub-milligram amounts, e.g., anisotropic NMR or microcrystal electron diffraction (Kunde and Schmidt, 2019; Liu et al., 2019), which facilitate the gathering of (new) natural products including eventual first bioactivity assays. However, the obtained quantities resulting from bioassay-guided isolation studies are, in general, far below the amounts necessary for clinical applications. Therefore, it is not surprising that the few reported trials applied essential oil (Caelli et al., 2000; Dryden et al., 2004; Edmondson et al., 2011; Blackwood et al., 2013) or extracts (Umashankar et al., 2018) rather than pure compounds (Rahim et al., 2021).

Though the so far conducted clinical trials were not convincing due to small sample sizes, missing randomization, or statistically insignificant results, they show a possible solution to overcome the problem of insufficient amounts of substance, namely the application of enriched (and standardized) extracts. As observed in this review, not seldom several constituents of a compound class and plant species exhibit positive effects, such as acetophenone sesquiterpenes, sphaerodiene diterpenes, and meroterpenes, which were isolated together with additional (less active) components of the same compound class. This complexity and the close structural similarity of the constituents hamper the isolation of the most active compounds and usually result in loss of substance. With the use of enriched extracts, preferably standardized to a certain (or minimum) concentration of active compound(s) or compound class, sufficient amounts could be provided for, e.g., topical formulations. Of course, when it comes to eventual further semi-synthetic drug optimization, pure substances will be needed. These optimizations not solely aim at potentiating the effect of a compound but are also needed to increase the compounds’ bioavailability and physical properties. For example, phenolic compounds, such as flavonoids, are rapidly eliminated, whereas many terpenoids show low solubility in aqueous solutions. Here, biotechnological approaches could guarantee provision of high-purity compounds that are produced in bioreactors. However, this requires understanding of the biosynthetic pathways leading to the natural product of interest, which currently is not the case for many plant species (Porras et al., 2021). Here, computational techniques such as genome mining and the use of genomic and transcriptomic databases show possible approaches to enable the synthesis of natural products in microorganisms. Alternatively, plant species such as *Nicotiana benthamiana* could serve as producing organisms, being more closely related and thus more suitable to create, e.g., glycosylated natural products (Molina-Hidalgo et al., 2021).

## 5. Summary and conclusion

In this review a total of 223 plant natural products from more than 20 different compound classes are presented and discussed. Though most of the reported compounds display moderate effects, with activities ranging from 10 to 50 μg/mL, a few exceptions act at low or even submicromolar concentrations. Two such compounds are the sesquiterpene lactones parthenolide (**24**) and lactupicrin (**25**), both showing a MIC value of 0.16 μg/mL. In addition, the subclass of acetophenone sesquiterpenes present a set of interesting candidates, with four compounds displaying pronounced effects (**26**–**28**, **31**). Similarly, the subclass of meroditerpenes, which also originate from two different biosynthetic pathways, yield several highly active constituents (**56**, **60**, **63**, **68**), with MIC values between 1.4 and 1.8 μg/mL. Further potent diterpenes were found in the class of abietanes (**48**, **49**) and sphaerodienes (**38**, **40**), respectively. Of note, for compound **38** MIC values of 0.25–1 μg/mL against five different strains have been determined. The class of triterpenoids only revealed one interesting compound (**80**), as did the class of alkaloids (**6**). However, many of the reported alkaloids displayed synergistic effects with existing antibiotics, which result from the inhibition of the bacterial efflux pump. (Table 1)

**Table 1:** Overview on additive and synergistic effects with existing antibiotics indicated by FICI values and combined MIC values, respectively.

No.	MRSA strain	Amikacin	Ampicillin	Azithromycin	Ceftazidime	Ciprofloxacin	Gentamicin	Levofloxacin	Norfloxacin	Oxacillin	Additional antibiotics	References
4	MRSA-011		*0.5*									Zeng et al. (2022)
	MRSA-003		*0.375*									
5	MRSA-011		*0.5*									Zeng et al. (2022)
	MRSA-003		*0.5*									
7	ATCC 33591										Ethidium bromide: 7.8 μg/mL (2×)	Zuo et al. (2011)
9	OMS 7		*0.625*							*0.5*		Yu et al. (2005)
	MRSA 004			*0.375*				*0.5*				Zuo et al. (2012)
	MRSA 055			*0.25*				*0.375*				
	MRSA 123			*0.25*				*0.375*				
	MRSA 144			*0.375*				*0.5*				
	MRSA 189			*0.375*				*0.5*				
	MRSA 240			*0.625*				*0.75*				
	MRSA 276			*0.375*				*0.5*				
	MRSA 294			*0.5*				*0.375*				
	MRSA 328			*0.25*				*0.375*				
	MRSA 330			*0.188*				*0.5*				
10	MRSA 004			*0.281*				*0.375*				Zuo et al. (2012)
	MRSA 055			*0.156*				*0.25*				
	MRSA 123			*0.375*				*0.188*				
	MRSA 144			*0.375*				*0.5*				
	MRSA 189			*0.5*				*0.188*				
	MRSA 240			*0.5*				*0.5*				
	MRSA 276			*0.375*				*0.5*				
	MRSA 294			*0.281*				*0.5*				
	MRSA 328			*0.5*				*0.375*				
	MRSA 330			*0.313*				*0.5*				
11	SA1199B								*0.375*			Yu et al. (2019)
46	ATCC 43300									0.25 μg/mL (256×)/*0.125*	Meropenem: *0.094*	Dettweiler et al. (2020)
											Vancomycin: *0.625*	
53	XU212										Tetracycline: 64 μg/mL (2×)	Oluwatuyi et al. (2004)
	RN4220										Erythromycin: 32 μg/mL (8×)	
	SA1199B								32 μg/mL (1×)		Ethidium bromide: 8 μg/mL (2×)	
54	XU212										Tetracycline: 32 μg/mL (4×)	Oluwatuyi et al. (2004)
	RN4220										Erythromycin: 256 μg/mL (1×)	
	SA1199B								32 μg/mL (1×)			
84	MRSA	*0.313–1*			*0.5–1*		*0.75–1.5*				Cefazolin: *0.625–2*	Zuo et al. (2015)
85	MRSA	*0.078–1*					*0.313–1*				Amoxicillin: *0.625–2*	Zuo et al. (2015)
90	T28.1					*0.49–1*	*0.14–1*			*0.28–0.75*	Rifampicin: *0.25–0.75*	Bocquet et al. (2019)
91	T28.1					*0.38–1.5*	*0.03–0.28*			*0.5–0.76*	Rifampicin: *1–5*	Bocquet et al. (2019)
94	MRSA	*0.25–2*					*0.25–1*				Etimicin: *0.375–0.75*	Zuo et al. (2014)
											Strepomycin: *0.25–1*	
95	MRSA			*0.47–0.75*	*0.188–2*		*0.375–1*				Penicillin*: 0.625–1*	Zuo et al. (2014)
96	MRSA	*0.625–1*			*0.5–1*		*0.75–1.5*				Cefazolin: *0.75–2*	Zuo et al. (2014)
108	MRA 004		*0.5*	*1.5*	*0.25*			*0.312*				An et al. (2011)
	MRA 055		*0.625*	*1*	*0.25*			*0.312*				
	MRA 092		*0.5*	*1*	*0.187*			*0.5*				
	MRA 123		*0.5*	*1*	*0.25*			*0.25*				
	MRA 144		*2*	*1*	*0.375*			*0.5*				
	MRA 155		*1*	*1.5*	*0.25*			*0.375*				
	MRA 189		*1*	*1.5*	*0.187*			*0.5*				
	MRA 247		*0.75*	*1.5*	*0.375*			*0.375*				
	MRA 328		*2*	*1*	*0.25*			*0.375*				
	MRA 330		*0.625*	*1.5*	*0.375*			*0.312*				
116	ATCC 43300					*0.16*	*0.13*			*0.14*	Tetracycline: *0.14*	Aelenei et al. (2020)
122	MRSA 1903									*0.625*		Navrátilová et al. (2016)
	MRSA 3202									*0.375*		
	MRSA 62097									*1.008*		
	MRSA 67755									*0.508*		
	MRSA 1679									*0.266*		
123	MRSA 1903									*0.266*		Navrátilová et al. (2016)
	MRSA 63718									*1.031*		
	MRSA 3202									*0.5*		
	MRSA 62097									*0.563*		
	MRSA 67755									*1.008*		
	MRSA 1679									*0.625*		
127	ATCC 43300					*0.38*	*0.19*			*0.27*	Tetracycline: *0.38*	Aelenei et al. (2020)
128	ATCC 29213						*0.135*					Meenu et al. (2021)
140	ATCC 1708										Meropenem: *0.375*	Kumarihamy et al. (2022)
											Vancomycin: *1*	
187	ATCC 33591		*0.5*							*0.5*		Lee et al. (2010)
	DPS-1		*0.5*							*0.5*		
	DPS-2		*0.5*							*0.5*		
	DPS-3		*0.5*							*0.5*		
	DPS-4		*0.37*							*0.5*		
	DPS-5		*0.5*							*0.5*		
	DPS-6		*0.5*							*0.75*		
	DPS-7		*0.5*							*0.5*		
	DPS-8		*0.5*							*0.5*		
	DPS-9		*0.37*							*0.5*		
	DPS-10		*0.5*							*0.5*		
	DPS-11		*0.37*							*0.5*		
	DPS-12		*0.37*							*0.5*		
	DPS-13		*0.37*							*0.5*		
	DPS-14		*0.5*							*0.37*		
	DPS-15		*0.37*							*0.5*		
190	T28.1					*0.63–1*	*9*			*0.19–1.25*	Rifampicin: *2.2–6*	Bocquet et al. (2019)

Strong synergistic effects (with ciprofloxacin, gentamicin, oxacillin, and tetracycline) were also reported for the prenylated flavones morusin (**116**) and kuwanon G (**127**). Likewise, taxifolin 7-O-α-L-rhamnopyranoside (**108**), displayed synergism with ceftazidime and levofloxacin, but was only slightly active alone. Three flavonol dicoumaroylrhamnosides (**112**–**114**), in contrast, exhibited pronounced effects with MIC values of 1.7–0.6 μg/mL. Other interesting phenolic compounds were found in the classes of isoflavonoids (**132**), xanthones (**170**, **171**), anthranoids (**187**, **191**), and above all, in the class of phloroglucinols. Here, several compounds exhibited remarkable effects, such as lupulone (**194**, MIC values of 0.6–1.2 μg/mL), ivesinol (**203**, MIC: 0.31 μg/mL), rhodomyrtone (**204**, MIC: 0.5 μg/mL), aspidinol (**205**, MIC: 0.5–2 μg/mL), and hyperforin (**206**, MIC: 0.5–2 μg/mL), thus rendering phloroglucinol derivatives the most promising compound class for further drug development. Finally, two rather unusual plant natural products have been reported, which were the cyclopentapyran **213** (with a MIC value of 1 μg/mL) and the macrolide neurymenolide A (**214**, with an IC_50_ value of 0.77 μg/mL).

In summary, several compounds with pronounced and/or synergistic effects against Methicillin-resistant *Staphylococcus aureus* have been isolated from plant species. Some of them even showed MIC values comparable to the established antibiotics vancomycin, daptomycin, tigecycline, and linezolid, respectively (Niveditha and Sujatha, 2015). Still, none of the mentioned compounds managed to proceed toward a commercial antibiotic drug. Whether the restricted activity to only a few out of several strains or their limited availability were the reason to not further develop the respective compounds could not be determined. In the latter case, the future will show if emerging biotechnological methods will help to provide higher amounts of natural products for subsequent clinical studies or semi-synthetic and technological modifications. In addition, more comprehensive approaches, e.g., studies with enriched extracts or defined fractions, may present an alternative for some stages in drug development. And last but not least, ethnopharmacological research will have to adapt to international conventions, e.g., the Nagoya protocol, taking them as what they are, namely measures to protect biodiversity and natural heritage and not obstacles in drug discovery.

## Author contributions

CMC conducted the literature search, organized the data, and wrote the original draft. SÇ was reviewing, writing, and editing the original draft, supervising the work, and acquiring the funding for publication. All authors contributed to the article and approved the submitted version.

## Funding

The authors acknowledged financial support by the DFG within the funding program “Open Access-Publikationskosten”.

## Conflict of interest

The authors declare that the research was conducted in the absence of any commercial or financial relationships that could be construed as a potential conflict of interest.

## Publisher’s note

All claims expressed in this article are solely those of the authors and do not necessarily represent those of their affiliated organizations, or those of the publisher, the editors and the reviewers. Any product that may be evaluated in this article, or claim that may be made by its manufacturer, is not guaranteed or endorsed by the publisher.
